# Perceived professional preparedness and identity among senior nursing students: a latent profile Analysis

**DOI:** 10.1186/s12912-024-01965-2

**Published:** 2024-04-29

**Authors:** Zuming Qin, Huilin Zhang, Siyu Su, Donghua Guo, Pei Wu, Yuting Huang, Huiping Wang

**Affiliations:** Clinical Nursing Teaching and Research Section, Second Xiangya Hospital, Central South University, Changsha, 410011 Hunan China

**Keywords:** Nursing students, Perceived preparedness, Professional identity, Latent profile analysis, Clinical preparation

## Abstract

**Background:**

Senior nursing students’ perceptions of their professional preparedness help them for expectations of their future nursing role with more confidence, and professional identity may contribute to cultivating nursing students’ perceptions of professional preparedness. In this study we applied latent profile analysis to identify the latent profiles of perceived professional preparedness among senior nursing students and to examine their identity and predictors.

**Methods:**

This was a cross-sectional descriptive study. A total of 319 senior nursing students from five universities in China were enrolled. Data were collected using the Perceived Professional Preparedness of Senior Nursing Students’ Questionnaire and the Professional Identity Scale for Nursing Students.

**Results:**

Three latent profiles were identified and labeled as “low perceived professional preparedness” (*n* = 90, 28.2%), “low clinical competency-low EBP (Evidence-Based Practice)” (*n* = 190, 59.5%), and “high perceived professional preparedness” (*n* = 39, 12.2%). Place of residence, average clinical practicum hours per day, part-time experience, good relationships with classmates, and feeling nobility toward nursing due to COVID-19 significantly predicted profile membership. The average professional identity score was also statistically different across the three profiles (*F* = 54.69, *p* < 0.001).

**Conclusions:**

Senior nursing students’ perceptions of their professional preparedness were divided into three profiles, and out results show that promoting professional identity may effectively foster their perceived professional preparedness. This study therefore highlights the importance of targeted interventions by considering their distinct perceptions of professional preparedness patterns.

## Introduction

Newly graduated nursing students are the main supply of annual increases in the nursing labor force [1]. However, these students frequently encounter difficulties during their transition to new clinical nurses. Challenges include adjusting to new ward environments, unfamiliar professional duties, experiencing role ambiguity [2], anxiety, and coping with physical and mental stress [3].A survey released by National Council of State Boards of Nursing (NCSBN) in 2020 showed that 25% of new nurses chose to leave in their first year of employment because of poor clinical transitions [4]. The lack of preparedness of newly graduated nurses to face their future careers has even become a global concern [5]. Clinical preparedness is defined as the capacity of nursing students to prioritize and deliver safe, high-quality care [6], and is believed to have a direct impact on the transformation to practice [7]. Research has found that senior nursing students with higher clinical preparedness tend to experience less anxiety when transitioning from school to work; they are more likely to adapt to the work environment [8] and are able to perform better in clinical practice [9].

Currently, there is no consensus on the definition of professional preparedness for nurses in clinical settings and it ignores nurses’ personal perceptions [7], which hinders the effective transition of nursing students into their professional roles. Additionally, many new graduates feel inadequately prepared and doubt their capabilities during this transition [10, 11]. Consequently, Shahsavari [12] proposed that the level of perceived preparedness should be regarded as a major output in all nursing education programs. Therefore, this study aims to assess senior nursing students’ levels of preparedness based on their perceptions and to explore the associated predictive factors of these levels.

## Background

Professional preparedness in nursing integrates the principles of nursing practice, nursing roles, and general skills (such as communication, critical thinking, and applying theory to practice) [13]. Perceived preparedness is a type of self-belief and confidence to be professionally competent [12]. The lack of perceived preparedness may therefore affect nurses’ confidence in their work and in communication with patients [14]. Recent research has found that the preparedness of nursing graduates can be motivated by part-time experience [15, 16], positive faculty-student and partner relationships [17], and adequate clinical exposure [18]. However, these studies overlook their perception of preparedness and mainly adopt variable-centered analysis methods that may ignore individual heterogeneity. To address these problems, latent profile analysis (LPA) may be appropriate. LPA is a person-centred approach that can identify subgroups of participants who share similar patterns based on variables of interest [19]. By using LPA, researchers can establish potentially different patterns of perceived professional preparedness among senior nursing students. Results from such analysis may help nursing educators tailor interventions to support nursing students in the future. Additionally, we explore the influence of senior nursing students’ demographics on their profiles, Prior research has shown that factors such as part-time experience, relationship with classmates, feeling a sense of belonging at a hospital, average clinical practicum hours per day, voluntary choice of nursing major and feeling nobility toward nursing due to COVID-19 could affect professional preparedness [15–18, 20–22]. In China, nursing students are required to complete an eight-month clinical practicum in general hospitals. Some of them, however, had to switch to online clinical practice due to COVID-19. Furthermore, the starting times of the clinical practice varied between schools, resulting in some students participating in online clinical practice for over a month, and others not participating at all. Therefore, the duration of online clinical practicum is also a factor we intend to explore.

Professional identity (PI) is another important factor we explored. PI includes the cognitive, emotional, and behavioral identification of nursing students within the nursing profession in which they will be working and within their current identities as nursing students [23]. As an important factor that determines to choose the nursing profession and is willing to actively learn [24], the importance of PI is self-evident. Previous studies have reported that a change in PI due to COVID-19 [21] and in addition have that improving nurses’ PI increases nurse retention in the workforce [25, 26]. PI has a significant positive impact on nursing students’ professional preparedness [20], but whether it has an exact impact on their perceptions of preparedness is uncertain. Given the potentially critical role of PI in improving perceived professional preparedness, its impact on different profiles needs to be studied as well.

What are the distinct pofiles of perceived professional preparedness among senior nursing students, and how do these pofiles relate to their professional identity and predictors of these perceptions? The study therefore aimed to identify perceived professional preparedness and identity among senior nursing students using LPA through (a) exploring potentially different profiles in perceived professional preparedness among senior nursing students; (b) identifying the characteristics of each profile; (c) comparing the PIs of latent profiles, thus providing targeted guidance for intervention to improve professional preparedness among senior nursing students. (d) identifying predictor of the latent profile of perceived professional preparedness.

## Methods

### Design

This study was a cross-sectional descriptive study, and its design and reporting were conducted in accordance with the guidelines for Strengthening the Reporting of Observational Studies in Epidemiology (STROBE).

### Participants

Firstly, we employed cluster random sampling among the cooperative universities of Second Xiangya Hospital. The random numbers were generated for each of the 30 cooperative universities by computer, and the five universities with the smallest random numbers were selected for the survey. Secondly, convenience sampling was applied to recruit volunteers for the questionnaire from the five universities. The eligibility criteria were: (1) full-time nursing students in their final year; (2) no prior work as a nurse; and (3) no cognitive or mental disorders.

### Sample size

Nylund-Gibson and Choi [27] recommend a minimum sample size of 300 cases for LPA in order to avoid problems in identifying smaller potential profiles. Additionally, we considered the general principles of sample size estimation for multivariate analysis to ensure the reliability of our subsequent multivariate analysis results: the number of observations should be at least 5 times the number of variables [28]. Taking into account a 20% dropout rate, we calculated that at least *N* = (11 + 19 + 17) * 5 / (1–20%) ≈ 293 participants are needed. Our final sample size was 319, meeting this minimum requirement.

### Data collection

The data were collected through anonymous and self-reported questionnaires. Prior to the informational survey, we conducted a pre-survey in March 2023 with 20 senior nursing students to assess whether the questions could be easily understood and whether any technical problems existed using an online self-reported questionnaire. The shortest response time was 150 seconds (these data are not included in this study). To ensure its reliability, our data collection instrument was piloted among 20 senior nursing students in March, 2023 to test its applicability, with a minimum response time of 150 seconds. We collected this data through an online Chinese questionnaire platform (www.wjx.cn) from April 6 to April 11, 2023. The questionnaire links were distributed using a convenience sampling method. An electronic poster was created to showcase the study’s purpose, significance, and inclusion criteria, and the corresponding author invited nursing students from five universities to participate by distributing the electronic poster and questionnaire link in a WeChat group. The collected questionnaires were then evaluated and those answered within 150 seconds were excluded from the analysis as they may not have been filled in carefully.

### Measurement

#### General information

A self-compiled online questionnaire was used to collect the individual characteristics for the latent profiles of perceived professional preparedness, including both demographic data (gender, age, place of residence, education level) and study-related information (average clinical practicum hours per day, online clinical practicum duration, voluntary choice of nursing major, part-time experience, relationship with classmates, feeling a sense of belonging at a hospital, feeling nobility toward nursing due to COVID-19).

#### Perceived professional preparedness of senior nursing students’ (PPPNS) questionnaire

The PPPNS questionnaire was compiled by Shahsavari [12] and translated into Chinese by Zhuo et al [29] This questionnaire has 19 items divided into four dimensions: clinical competency (5 items), evidence-based practice (5 items), framework-oriented performance (4 items), and patient-centered care (5 items). Each item is scored from 1 to 5 on a Likert scale (1 = completely disagree; 5 = completely agree), and the total score thus ranges from 19 to 95, with a higher score indicating better perceived professional preparedness. The Cronbach’s alpha of the Chinese version of the PPPNS is 0.977, and in this study, the Cronbach’α was 0.942. The correlation coefficients between the total score and each item were also between 0.81 and 1.00(*P* < 0.01), and the content validity index was 0.90 (CVI ≥ 0.80). In addition, the test-retest reliability was 0.893, indicating good reliability and validity.

#### Professional identity

The Professional Identity Scale for Nursing Students (PISNS) measures PI. Developed by Hao et al. [30], it includes 17 items in five dimensions: professional self-image, the benefit of retention and risk of turnover, social comparison and self-reflection, independence of career choice, and social modeling. All items in the PISNS scale are scored from 1 (strongly disagree) to 5 (strongly agree) on a Likert scale, and item 12 is scored in reverse. The scale has a maximum score of 85, with higher scores indicating higher levels of PI. Construct validity showed five factors model explaining 58.9% of the total variance. Content validity of the questionnaire were assessed by 4 experts. They examined the clarity and simplicity of all items. The test–retest reliability, Cronbach’s alpha, and split-half reliability of the scale were 0.74, 0.83, and 0.84, respectively, indicating good reliability and validity.

### Data analysis

IBM SPSS 25.0 and Mplus 8.3 were used to analyze the data. The tests described below were all two-sided, with *p* < 0.05 indicating statistically significant results.

#### LPA

An exploratory LPA was conducted using Mplus 8.3 to examine the latent profiles of perceived professional preparedness among senior nursing students. Five models, ranging from the initial (1 profile) to the final (5 profiles), were estimated by gradually increasing the number of profiles until the fitness indices had achieved the optimal level. To identify the optimal number of profiles, we used the Bayesian Information Criterion (BIC), the Akaike Information Criterion (AIC), and the sample-size adjusted BIC (aBIC), with smaller values indicating better model fit [31]. Entropy values were also calculated, with an entropy value closer to 1.0 indicating greater precision of classification [31]. In addition, the *p* values calculated by the Lo–Mendell–Rubin test (LMR) and bootstrap likelihood ratio test (BLRT) are crucial metrics for determining whether a model best suits the data [31]. The *p*-value < 0.05 indicated that the model fits the data significantly better than the previous model [32]. When selecting the optimal model, we also considered model parsimony (favoring less complex models) and the size of the profiles (at least 5% of the total sample to exclude non-replicable profiles). We evaluated each solution’s meaningful distinctiveness, ensuring profiles differed qualitatively, not just quantitatively [33], to add new, significant information to the model.

#### Multinomial logistic regression analysis and one-way analysis of variance

After selecting the optimal model, a multinomial logistic regression analysis was performed in SPSS 25.0 to explore the predictors of profile membership, and the differences in the PI scores in each latent profile were obtained using one-way analysis of variance and the Student–Newman–Keuls (SNK) test.

#### Common method bias test

The data collection for this study was done in the same context, and this may have introduced common method bias [34]. We therefore used Harman single-factor test analysis for all PPPNS and PISNS items in SPSS 25.0. If at least two common factors are found and the variance explanation rate of the first does not exceed 40%, then there is no common method bias [35]. Our results showed that 6 common factors were present, and the rate of the first common factor was 39.84% (< 40%). Thus, no common method bias was deemed to be present.

## Ethics

This study was approved by the Institutional Review Board of Nursing and Behavioral Medicine Research, School of Nursing, Central South University (approval NO. E202361). An online informed consent form was presented on the homepage of the online questionnaire, and all participants were informed that their participation was voluntary and confidential and that they could withdraw from the study at any time for any reason and without facing negative or disciplinary consequences of any kind.

## Results

A total of 338 electronic questionnaires were distributed, and 319 were collected, for a validity rate of 94.37%. The survey involved mostly female senior nursing students (89.3%) with a mean age of 20.56 years (*SD* = 1.219). As for education, 252 were junior college students (79.0%), and 67 were undergraduates (21.0%). Most of the participants came from rural areas (63.0%), followed by towns (20.7%), and urban areas (16.3%). Less than one-sixth of the senior nursing students came from single-child households. Furthermore, over 50% of the participants had part-time nursing experience in school.

### Latent profiles of perceived professional preparedness

Table 1 shows the fit metrics for five estimated models. The three-profile model had the lower Log(L), AIC, BIC, and aBIC values than the two-profile model, and the highest Entropy value (0.967). Furthermore, the LMR value of the four-profile model was not significant, indicating that the three-profile model was better. The optimal fit metrics are highlighted in bold in Table 1.

**Table 1:** Fit metrics of each model

Model	k	Log(L)	AIC	BIC	aBIC	Entropy	LMR	BLRT
1-profile	38	− 6239.878	12,555.756	12,698.834	12,578.305	–	–	–
2-profile	58	− 5364.686	10,845.373	11,063.754	10,879.789	0.922	0.016	< 0.000
**3-profile**	**78**	**− 4887.933**	**9931.867**	**10,225.552**	**9978.151**	**0.967**	**0.018**	**< 0.000**
4-profile	98	− 4683.588	9563.177	9932.166	9621.329	0.955	0.098	< 0.000
5-profile	118	− 4526.090	9288.180	9732.472	9358.199	0.965	0.587	< 0.000

*k* Number of free parameters, *Log(L)* Log-likelihood value, *AIC* Akaike information criterion, *BIC* Bayesian information criteria, *aBIC* adjusted Bayesian information criteria, *LMR* Lo–Mendell–Rubin Test, *BLRT* Bootstrap Likelihood Ratio Test

**Fig. 1:**
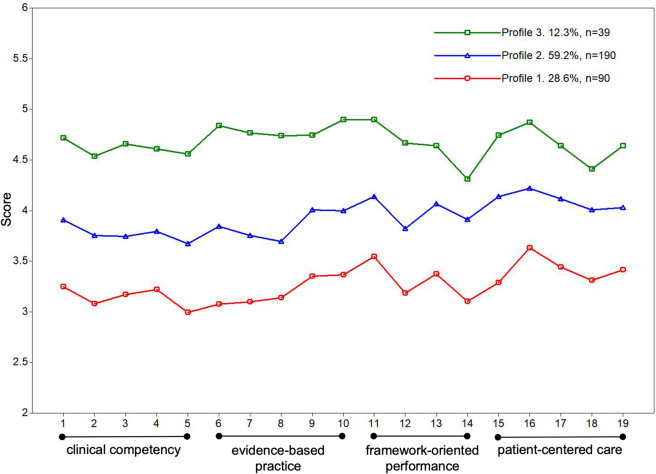
Latent profiles of perceived professional preparedness of senior nursing students

The scores for the four dimensions of the three profiles and their 19 items are presented in Fig. 1. Profile 1, comprising 28.2% of participants (*n* = 90) and named the “low perceived professional preparedness” group, reported the lowest scores for all items. Profile 2 was the “low clinical competency-low EBP” group and accounted for 59.5% (*n* = 190), with scores lower than Profile 3, especially in “clinical competence” dimension and the “evidence-based practice” dimension. Profile 3, named the “high perceived professional preparedness” group, accounted for 12.2% of participants (*n* = 39), scored highest on all PPPNS items.

### Demographic and study-related characteristics of each profile

The demographic and study-related characteristics of the participants are shown in Table 2.

**Table 2:** Demographic and study-related features by latent profile membership, *n* (%)

	Overall (*n* = 319)	Profile 1 (*n* = 90)	Profile 2 (*n* = 190)	Profile 3 (*n* = 39)
Gender				
Male	34 (10.7)	12 (13.3)	18 (9.5)	4 (10.3)
Female	285 (89.3)	78 (86.7)	172 (90.5)	35 (89.7)
Age				
≤ 19	56 (17.6)	24 (26.7)	25 (13.2)	7 (17.9)
20–21	196 (61.4)	51 (56.7)	119 (62.2)	26 (66.7)
>22	67 (21.0)	15 (16.7)	46 (24.2)	6 (15.4)
Place of residence				
Rural	54 (16.9)	16 (17.8)	25 (13.2)	13 (33.3)
Town	68 (21.3)	19 (21.1)	36 (18.9)	13 (33.3)
Urban	197 (61.8)	55 (61.1)	129 (67.9)	13 (33.3)
Education level				
Associate degree	252 (79.0)	73 (81.1)	149 (78.4)	30 (76.9)
Bachelor’s degree	67 (21.0)	17 (18.9)	41 (21.6)	9 (23.1)
Average clinical practicum hours per day				
<7	27 (8.5)	17 (18.9)	7 (3.7)	3 (7.7)
7–9	264 (82.8)	65 (72.2)	166 (87.4)	33 (84.6)
>9	28 (8.8)	8 (8.9)	17 (8.9)	3 (7.7)
Online clinical practicum duration (week)				
<1	148 (46.4)	53 (58.9)	73 (38.4)	22 (56.4)
1–4	104 (32.6)	21 (23.3)	72 (37.9)	11 (28.2)
>4	67 (21.0)	16 (17.8)	45 (23.7)	6 (15.4)
Voluntary choice of the nursing major				
Yes	253 (79.3)	65 (72.2)	155 (81.6)	33 (84.6)
No	66 (20.7)	25 (27.8)	35 (18.4)	6 (15.4)
Part-time experience				
Yes	164 (51.4)	35 (38.9)	103 (54.2)	26 (66.7)
No	155 (48.6)	55 (61.1)	87 (45.8)	13 (33.3)
Good relationships with classmates				
Yes	296 (92.8)	74 (82.2)	184 (96.8)	38 (97.4)
No	23 (7.2)	16 (17.8)	6 (3.2)	1 (2.6)
Feeling a sense of belonging at a hospital				
Yes	249 (78.1)	61 (67.8)	152 (80.0)	36 (92.3)
No	70 (21.9)	29 (32.2)	38 (20.0)	3 (7.7)
Feeling nobility toward nursing due to COVID-19				
Yes	246 (77.1)	57 (63.3)	154 (81.1)	35 (89.7)
No	73 (22.9)	33 (36.7)	36 (18.9)	4 (10.3)

### Predictors of latent profile membership

A multinomial logistic regression analysis was carried out with reference to the Profile 1 group in order to pinpoint the variables connected to the three profiles. Compared to the Profile 1 group, senior nursing students who worked less than 7 hours per day during the clinical practicum (*OR* = 0.218, *P* = 0.031) were less likely to be in the Profile 2 group. Whereas those residing in town (*OR* = 5.346, *P* = 0.006) and urban areas (*OR* = 3.413, *P* = 0.028), and having part-time experience were more possibly to be in the Profile 3 group. Moreover, senior nursing students who had good relationships with classmates and felt nobility toward nursing due to COVID-19 were more likely to belong to Profiles 2 (*OR* = 6.917/2.717, *P* = 0.002/0.008) and 3 (*OR* = 11.403/4.540, *P* = 0.047/0.031) Table 3.

**Table 3:** Predictors of latent profile membership

	B	SE	OR	95% confidence interval	*P*
**Profile 2 (vs. Profile 1)**					
Gender (ref: Male)					
Female	0.074	0.543	1.077	0.372–3.120	0.891
Age (ref: >22)					
18–19	−0.826	0.554	0.438	0.148–1.297	0.136
>22	−0.413	0.459	0.662	0.269–1.628	0.369
Place of residence (ref: Rural)					
Urban	−0.193	0.446	0.824	0.344–1.976	0.665
Town	−0.087	0.384	0.917	0.432–1.944	0.820
Education level (ref: Bachelor’s degree)					
Associate degree	0.713	0.591	2.041	0.640–6.503	0.228
Average clinical practicum hours per day (ref: >9)					
<7	−1.525	0.708	0.218	0.054–0.872	0.031*
7–9	−0.090	0.516	0.914	0.332–2.512	0.861
Online clinical practicum duration (week, ref.: >4)					
<1	−0.607	0.431	0.545	0.234–1.268	0.159
1–4	−0.076	0.456	0.927	0.380–2.265	0.868
Voluntary choice of the nursing major (ref: No)					
Yes	0.044	0.402	1.045	0.475–2.297	0.913
Part-time experience (ref: No)					
Yes	0.467	0.317	1.596	0.857–2.973	0.141
Good relationships with classmates (ref: No)					
Yes	1.934	0.610	6.917	2.092–22.868	0.002*
Feeling a sense of belonging at a hospital (ref: No)					
Yes	−0.178	0.388	0.837	0.391–1.792	0.647
Feeling nobility toward nursing due to COVID-19 (ref: No)					
Yes	0.999	0.374	2.717	1.304–5.659	0.008*
**Profile 3 (vs. Profile 1)**					
Gender (ref: Male)					
Female	−0.356	0.788	0.701	0.150–3.279	0.651
Age (ref: >22)					
18–19	−0.071	0.893	0.931	0.162–5.359	0.936
>22	0.179	0.710	1.196	0.297–4.808	0.801
Place of residence (ref: Rural)					
Urban	1.676	0.607	5.346	1.625–17.579	0.006*
Town	1.228	0.558	3.413	1.144–10.184	0.028*
Education level (ref: Bachelor’s degree)					
Associate degree	0.260	0.824	1.296	0.258–6.522	0.753
Average clinical practicum hours per day (ref: >9)					
<7	−0.480	1.100	0.619	0.072–5.348	0.663
7–9	0.164	0.825	1.178	0.234–5.932	0.842
Online clinical practicum duration (week, ref: >4)					
<1	0.618	0.673	1.855	0.496–6.940	0.359
1–4	−0.101	0.705	0.904	0.227–3.601	0.886
Voluntary choice of the nursing major (ref: No)					
Yes	0.433	0.646	1.542	0.435–5.463	0.503
Part-time experience (ref: No)					
Yes	0.970	0.482	2.638	1.026–6.784	0.044*
Good relationships with classmates (ref: No)					
Yes	2.434	1.226	11.403	1.031–126.141	0.047*
Feeling a sense of belonging at a hospital (ref: No)					
Yes	0.915	0.745	2.498	0.580–10.762	0.219
Feeling nobility toward nursing due to COVID-19 (ref: No)					
Yes	1.513	0.700	4.540	1.152–17.892	0.031*

### PI with latent profile membership

Analysis of variance was performed to explore the difference in PI among the three profiles, as shown in Table 4. The mean scores of the PI of senior nursing students in Profiles 1, 2, and 3 were 58.92 (*SD* = 7.55), 66.18 (*SD* = 6.70), and 74.18 (*SD* = 6.98), respectively, and their five dimensions statistically differed across the three profiles (*p* < 0.001). Additionally, the SNK test revealed that the mean score of the “high perceived professional preparedness” group was the highest, while the “low perceived professional preparedness” group were the lowest.

**Table 4:** PI difference between the three profiles, M ± SD

	Profile 1 (*n* = 90)	Profile 2 (*n* = 190)	Profile 3 (*n* = 39)	F	*P*	SNK
Professional identity	58.92 ± 7.558	66.18 ± 6.705	74.18 ± 6.984	54.693	< 0.001	3 > 2 > 1
Professional self-image	20.43 ± 3.474	23.53 ± 3.109	26.59 ± 3.314	47.292	< 0.001	3 > 2 > 1
Benefit of retention and risk of turnover	13.36 ± 2.290	15.16 ± 1.970	17.08 ± 2.157	55.820	< 0.001	3 > 2 > 1
Social comparison and self-reflection	10.96 ± 1.513	12.02 ± 1.186	13.54 ± 1.274	8.764	< 0.001	3 > 2 > 1
Independence of career choice	6.50 ± 1.104	6.99 ± 1.254	7.46 ± 1.668	49.765	< 0.001	3 > 2 > 1
Social modeling	7.57 ± 1.152	8.47 ± 1.062	9.51 ± 0.721	70.358	< 0.001	3 > 2 > 1

## Discussion

### Latent profiles of perceived professional preparedness

This study is the first to use LPA to determine the underlying profile of senior nursing students’ perceived professional preparedness, thus augmenting previous studies that treat senior nursing students as a homogeneous group. Such an approach may well guide further research on tailored interventions to improve nursing students’ perceived preparedness. Based on the responses to each item in the PPPNS questionnaire, three subgroups were identified, the “low perceived professional preparedness”, “low clinical competency-low EBP”, and “high perceived professional preparedness” groups. The scores of the PPPNS questionnaire (72.78 ± 9.02) in our study were significantly higher than Xu et al. [20] (64.59 ± 10.29). A possible reason for this difference is that the study by Xu et al. concentrated on nursing students who were yet to finish their clinical practicum, resulting in less professional preparedness. Conversely, our study involved senior nursing students who had already completed their clinical practicum.

The “low perceived professional preparedness” group comprised the 28.2% of senior nursing students who scored lowest in all dimensions, indicating that they perceived their upcoming transitions to a clinical setting poorly and lacked confidence in their clinical competence. The reason might be that they feel difficulty in bridging the gap between theory and practice in preparing for clinical nursing [36]. If this is the case, we suggest that nurse educators should adopt measures to support senior nursing students in transitioning to clinical environments like simulated clinical experience (SCE) [37]. Researchers highlight that the type of support strategy is less important. What matters is the organization’s focus on and investment in their transition [38].

The “low clinical competency-low EBP” group was the largest subgroup, accounting for 59.5% of the total. Compared to the “high perceived professional preparedness” group, the senior nursing students in this group felt that their clinical and evidence-based practice competence was relatively insufficient, which is likely a problem faced by most senior nursing students. Clinical competence is the foundation of nursing practice and is directly related to college education [39]. In Australia, for example, students begin clinical practice in the first semester after their enrollment, and this hands on practice is well-integrated with theoretical classroom study [40]. However, in China the clinical practicum is mostly conducted by observation and occurs in the last academic year [41]. Evidence-based practice (EBP) is considered to be the core method of bridging the gap between one’s own knowledge and current knowledge in nursing practice [42], and research has shown that nursing students generally have a low awareness of evidence-based practice but have a positive attitude [43]. Moreover, nursing students who acquire evidence-based practical knowledge mainly from the classroom have poorer poor learning results. Du et al. [44] incorporated EBP elements into nursing research courses based on Astin’s Input-Environment-Outcome model and found that it effectively improved nursing students’ EBP. It suggests that it is important for nursing educators to seek educational program reforms in this case.

The “high perceived professional preparedness” group, which constituted 12.2% of the sample, had the highest scores on the PPPNS items. These senior nursing students believe that they are competent for future clinical work. This subgroup perceived professional preparedness significantly higher than the other two groups, and the reason deserves further exploration. The 14th item, “I think I can earn the trust of patients and their families”, had the lowest score in this group, however, indicating that they still expected it to be it hard to gain trust from patients and their families even though they perceived themselves to be able to provide good care. This may be related to inadequate nurse-patient communication training or feelings of incompetence in caring for multiple patients at the same time [45].

### Demographic and study-related characteristics of each profile

One of the demographic predictors of profile membership was residence. In China, there is a large gap in basic education between urban and rural areas. Residents in rural areas generally have a low level of education, and fewer educational resources [46]. On the contrary, those who live in towns and urban areas enjoy richer learning resources and better learning environments, which can support them in obtaining better educations. Studies have also found that nursing students living in rural areas have lower subjective well-being [47]. It indicates that organizations should be aware of the impact of this factor on nursing students’ professional preparedness.

Our study-related predictors of profile membership included average clinical practicum time, relationship with classmates, part-time experience, and feelings of nobility toward nursing due to COVID-19. Compared to the average practice time of more than 9 hours per day, nursing students whose practice time was ≤7 hours were unlikely to be in the “low clinical competency-low EBP” group. In China, nursing students need to practice for an average of 8 hours per day. But due to COVID-19 in some areas, they were required to leave work early, making their daily practice time shorter. Insufficient internship duration has led to inadequate professional preparedness. According to our results, although clinical practicum duration cannot be considered to have had a direct impact on perceived professional preparedness, a duration of less than 8 hours per day was still a predictor of poor preparedness. This aligns with the research by Monir Almotairy [22].

In addition, those senior nursing students who had good relationships with classmates and feelings of nobility toward nursing due to COVID-19 were more likely to be in Profile 2 and Profile 3. Being able to get along well with classmates reflects good interpersonal communication skills, and is one of the core competencies of nursing [48], developing good communicative competence enables nursing students to adapt to the clinical environment and maintain a good rapport with patients, thus improving their confidence in clinical practice [49]. During COVID-19, a large number of excellent nurses stuck to the “frontline” of treatment, which inspired a certain professional honor for nursing students themselves and largely girded their resolve to enter the profession [21]. This finding was also observed in the study by Li et al. [50] It suggests that the cultivation of a sense of professionalism may play a positive role in enhancing nurses’ perception of their professional preparedness.

Next, we found that nursing students with part-time experience were more likely to be in the group with “high perceived professional preparedness”, which is consistent with the findings of several studies [15, 16]. Part-time experience can help college students better adapt to their careers and demonstrate a greater appreciation for developing positive psychological capital [51]. Studies on nursing students have also shown that nursing students with part-time experience have higher career adaptability than those without such experience [52]. This indicates that part-time experience can facilitate the transition to new environments and future careers. Therefore, nursing educators should recognize the positive impacts of part-time experience on nursing students and seek to implement them wherever possible.

### PI of the each profile

In this study, the PI score (65.11 ± 8.37) was higher than the scores reported in the national surveys by Zhang [53] and Tang [54] in 2020, which were 62.02 ± 12.02 and 59.49 ± 12.41, respectively. MacIntosh [55] stated that professional identity is a developing process influenced by professional socialization. 2020 was the initial phase of the COVID-19 outbreak in China, whereas our study was conducted during a period of comprehensive reopening. Over time, there has been a gradual and more positive shift in nursing students’ perceptions of the profession [56]. However, there may also be a bias due to the smaller scope of sample collection in our study.

The average PI score of the “high perceived professional preparedness” group was significantly higher than that of the other two groups, indicating that nursing students with higher PI scores were more confident about their professional preparedness. Therefore, nursing students with a strong PI have a positive perception and evaluation of the nursing profession. They have a clearer understanding of the meaning and purpose of nursing, which motivates them to attach importance to the cultivation of professional skills and to study hard in order to achieve success in their chosen profession [57]. This suggests that nursing educators can enhance nursing students’ PI through activities that inspire their sense of professional honor such as showcasing nursing role models [57]. However, there is no research on whether the improvement in PI correspondingly affects the perceptions of professional preparedness of nursing students or on its impact at different time periods in education. Exploration of this seems to hold much promise. In conclusion, promoting PI may be an effective way to cultivate senior nursing students’ perceived professional preparedness.

### Implications and limitations

When developing interventions to improve the professional preparedness of nursing students, nursing educators should explore effective ways to integrate theoretical knowledge with clinical practice. For example, they could achieve this by utilizing the flipped classroom approach, engaging in clinical scenario simulation exercises for multiple patient care [37], and/or reforming clinical practicum requirements. Moreover, it is also crucial to improve nursing students’ information literacy. Nursing schools should therefore provide students with expanded access to information and sufficient learning resources to support their EBP development.

This study also has some limitations, however. First, limited by the study’s cross-sectional design, we were not able to explore the causal effect of the perceptions of professional preparedness. Therefore, longitudinal studies should be the focus of future research. Second, as our participants came from only two provinces of China, a multi-center sample is needed in the future in order to make the results more representative and reliable. Finally, the participants from different regions were affected differently by COVID-19, so our study results cannot definitively determine its effect on them using the specified models.

## Conclusion

This study used LPA to find three latent profiles in perceived professional preparedness among senior nursing students in China, involving the “low perceived professional preparedness” group, the “low clinical competency-low EBP” group, and the “high perceived professional preparedness” group. Place of residence, average clinical practicum hours per day, part-time experience, good relationships with classmates, and feeling nobility toward nursing due to COVID-19 significantly predicted profile membership. Further comparison of the PI for each profile showed that the “high perceived professional readiness” group had a significantly higher average PI score than the other two groups. We conclude that targeted interventions should be formulated based on student demographics and study-related characteristics for each profile. Promoting professional identification may also be effective in promoting the perception of professional preparedness.

## Data Availability

The datasets used and/or analysed during the current study are available from the corresponding author on reasonable request.

## Abbreviations

COVID-19: Coronavirus disease 2019
PPPNS: Perceived professional preparedness of senior nursing students
PI: Professional identity
PISNS: Professional identity scale for nursing students
LPA: Latent profile analysis
Log(L): Log-likelihood value
AIC: Akaike information criterion
BIC: Bayesian information criterion
aBIC: Adjusted Bayesian information criterion
LMR: Lo–mendell–rubin test
BLRT: Bootstrap likelihood ratio test
SNK: Student–newman–keuls
SD: Standard deviation
EBP: Evidence-based practice
